# Correlation between Peripheral Levels of Brain-Derived Neurotrophic Factor and Hippocampal Volume in Children and Adolescents with Bipolar Disorder

**DOI:** 10.1155/2015/324825

**Published:** 2015-05-13

**Authors:** Tatiana Lauxen Peruzzolo, Mauricio Anes, Andre de Moura Kohmann, Ana Claudia Mércio Loredo Souza, Ramiro Borges Rodrigues, Juliana Basso Brun, Roberta Peters, Bianca Wollenhaupt de Aguiar, Flavio Kapczinski, Silzá Tramontina, Luis Augusto Paim Rohde, Cristian Patrick Zeni

**Affiliations:** ^1^Pediatric Bipolar Disorder Outpatient Program (ProCAB), Universidade Federal do Rio Grande do Sul (UFRGS), 90035-903 Porto Alegre, RS, Brazil; ^2^Hospital de Clínicas de Porto Alegre, Division of Medical Physics and Radiation Protection, 90035-903 Porto Alegre, RS, Brazil; ^3^Bipolar Disorder Unit, Molecular Psychiatry Unit and National Institute for Translational Medicine, CNPq., 90035-903 Porto Alegre, RS, Brazil; ^4^Federal University, UFRGS, 90035-903 Porto Alegre, RS, Brazil; ^5^UTHealth, Houston, TX 77030, USA; ^6^ADHD Outpatient Program (PRODAH), UFRGS, 90035-903 Porto Alegre, RS, Brazil; ^7^National Science and Technology Institute for Children and Adolescents, 05403-010 São Paulo, SP, Brazil; ^8^University of Texas Health Science Center, Houston, TX 77030, USA

## Abstract

Pediatric bipolar disorder (PBD) is a serious mental disorder that affects the development and emotional growth of affected patients. The brain derived neurotrophic factor (BDNF) is recognized as one of the possible markers of the framework and its evolution. Abnormalities in BDNF signaling in the hippocampus could explain the cognitive decline seen in patients with TB. Our aim with this study was to evaluate possible changes in hippocampal volume in children and adolescents with BD and associate them to serum BDNF. Subjects included 30 patients aged seven to seventeen years from the ProCAB (Program for Children and Adolescents with Bipolar Disorder). We observed mean right and left hippocampal volumes of 41910.55 and 41747.96 mm^3^, respectively. No statistically significant correlations between peripheral BDNF levels and hippocampal volumes were found. We believe that the lack of correlation observed in this study is due to the short time of evolution of BD in children and adolescents. Besides studies with larger sample sizes to confirm the present findings and longitudinal assessments, addressing brain development versus a control group and including drug-naive patients in different mood states may help clarify the role of BDNF in the brain changes consequent upon BD.

## 1. Introduction

Bipolar disorder (BD) is a severe mental disorder characterized by mood swings during which a person has distinct periods of impairing elevated (mania) or decreased (depression) mood and energy [1]. It occurs in approximately 0.4 to 1.6% of adults and in 1% in children and adolescents [2, 3]. In the early-age onset presentation (pediatric bipolar disorder, PBD), difficulties in interpersonal relationships, academic functioning, and negative outcomes such as multiple hospitalizations and high rates of suicide attempts are observed [4, 5].

Despite the devastating effects of BD on child development, little is known about the causes of this disorder. Its etiology is probably multifactorial, including biological and environmental factors [6]. Studies in adults with BD suggest that neurotrophins, particularly brain-derived neurotrophic factor (BDNF), inflammatory markers, and oxidative stress may be related to the etiology of this disorder [7, 8]. BDNF is the most abundant neurotrophin in the brain, and it has been implicated in neuronal processes such as neurogenesis, neuronal survival, dendritic growth, and synaptic plasticity [9]. It has been suggested that neuronal viability might be affected by neurotrophins persistent reduction [10]. Kauer-Sant'Anna and colleagues found BDNF levels were lower in patients who had multiple episodes of the disorder, which led to the hypothesis that episode-related reduction of neurotrophins could explain some of the structural changes in the brain observed in bipolar patients [11]. Besides that, BDNF is highly expressed in the cortex and hippocampus, areas of the brain known to regulate complex functions such as memory and emotion.

Structural and functional neuroimaging studies of pediatric BD generally converge with adult studies in implicating frontolimbic structures [12] and smaller sizes of amygdala [13] and hippocampus [14, 15], and a significant negative correlation between the volume of the right hippocampus of adolescents with BD and disease duration has been reported. Although these findings are preliminary due to relatively small sample sizes, hippocampal commitment is consistent across several investigations.

Altogether, these findings suggest that abnormalities of BDNF signaling in the hippocampus could be an explanation to the cognitive deficits observed in PBD and brain alterations present in adults after multiple episodes [10, 16]. The most consistent associations between PBD and cognitive deficits were reported for impairments in working memory, verbal memory, attention, executive function, response flexibility, reversal learning, processing speed, set shifting, and visuospatial memory [17].

Due to the involvement of BDNF in BD and its abundance and influence on neurogenesis in the hippocampus, we evaluated the correlation between peripheral levels of BDNF and hippocampal volumetric measurements in children and adolescents with BD. Furthermore, based on previous studies showing [1] early cognitive deficits in PBD, we evaluated the working memory of patients with PBD. We also hypothesized that patients with longer disease duration would present lower serum BDNF levels, poorer neurocognitive performance, and reduced hippocampal volumes.

## 2. Methods

This was a cross-sectional study. Children and adolescents with bipolar disorder I, bipolar disorder II, or bipolar disorder NOS evaluated in the ProCAB (Pediatric Bipolar Disorder Outpatient Program) of the Hospital de Clínicas de Porto Alegre were invited to participate. Enrollment was performed from 2012 to 2013. Inclusion criteria were as follows: ages 7–17 years; both genders; bipolar diagnosis I, bipolar diagnosis II, or bipolar diagnosis NOS according to DSM-IV. Of note, since the most representative sample of pediatric bipolar disorder is the COBY study (Course and Outcome of Bipolar Youth), our definition BD-NOS followed the same criteria, that is, at least 4 episodes of 4-hour lasting mood changes clearly differing from the usual for the subject [18]. Exclusion criteria were as follows: presence of schizophrenia, pervasive developmental disorder, active substance abuse, and contraindications to MRI.

### 2.1. Diagnostic Assessment

All patients underwent a three-step procedure for diagnosis ascertainment.

First, a child and adolescent psychiatrist performed BD symptom (DSM-IV and DSM5 criteria) and family history of mental disorders screening with parents and children together. A total of 127 subjects were assessed, and 95 (75%) were excluded due to not presenting BD-I, BD-II, or BD-NOS. Two patients started the assessment but did not complete it.

When the diagnosis of BD was suspected, patients and parents went through a semistructured interview with the schedule for affective disorders and schizophrenia for school-age children, present and lifetime version (K-SADS-PL), conducted by a research assistant.

Finally, a clinical evaluation was conducted by a second child and adolescent psychiatrist that received all information from previous queries. An important observation is that a clinical meeting with all the professionals involved in the assessment was conducted to define diagnosis, comorbidity, and the treatment plan.

Patients diagnosed with BD-I, BD-II, or BD-NOS underwent neuropsychological assessment, blood sampling for BDNF level determination, and MRI. Young Mania Rating Scale (YMRS) and Children's Depression Rating Scale (CDRS) were also applied for the purpose of measuring manic and depressive symptoms at the evaluation time.

### 2.2. Neuropsychological or Neurocognitive Assessment

Full scale IQ was determined using the vocabulary and block design subsets of the Wechsler Intelligence Scale for Children-Third Version (WISC-III) and the Digit Span of the WISC-III (total score and inverse order). According to previous studies, Digit Span Inverse Order is more directly correlated with working memory [19].

### 2.3. Neuroimaging Assessment

#### 2.3.1. Images Acquisition

All patients underwent a 1.5 T Philips Achieva magnetic resonance imaging (MRI) using an eight-channel head coil. The structural images were acquired using a sagittal 3D T1 weighted magnetization-prepared rapid gradient-echo (MPRAGE) sequence (repetition time = 8.7 ms, echo time = 4.0 ms, inversion time = 1000 ms, and flip angle = 8°). Possible head movements were minimized by placing foam pads inside the head coil. The volumetric segmentation and measurement were performed with the FreeSurfer image analysis suite, which is documented and freely available for download online (http://surfer.nmr.mgh.harvard.edu/).

#### 2.3.2. Processing of Structural Images

The image processing was carried out with support from the National Supercomputing Center (CESUP), Federal University of Rio Grande do Sul [20]. FreeSurfer v 5.3 was installed in the cluster with Novell SUSE Linux Enterprise Server 11-SP1 operating system. Each data set subject was allocated to one processing core and included in the submission queue processing script.

Cortical reconstruction and volumetric segmentation were performed with the FreeSurfer image analysis suite, which is documented and freely available for download online (http://surfer.nmr.mgh.harvard.edu/). Briefly, this processing includes motion correction and averaging of multiple volumetric T1 weighted images (when more than one is available), removal of non-brain tissue using a hybrid watershed/surface deformation procedure, automated Talairach transformation, segmentation of the subcortical white matter, and deep gray matter volumetric structures (including hippocampus, amygdala, caudate, putamen, and ventricles) [21, 22]. Automatic segmentation method was used to measure hippocampal volume. Shortly, a neuroanatomical label is assigned to every voxel in the brain, comparing to an atlas whose encoding is based on class and location classification. This procedure is based on modeling the segmentation as a nonstationary anisotropic Markov random field (MRF), in which the probability of a label is modulated by the probability of its neighbors, with the probabilities computed separately at each position in an atlas, for each pair of tissue classes and for each of the six cardinal directions [21].

### 2.4. BDNF Serum Levels Determination

BDNF serum levels were measured with sandwich-ELISA, using a commercial kit according to the manufacturer's instructions (Millipore, USA). Briefly, microtiter plates (96-well flat-bottom) were coated for 24 h at 4°C with the samples diluted 1 : 100 in sample diluent and standard curve ranging from 7.8 to 500 pg of BNDF. Plates were then washed four times with wash buffer followed by the addition of biotinylated mouse anti-human BNDF monoclonal antibody (diluted 1 : 1000 in sample diluent), which was incubated for 3 h at room temperature. After washing, a second incubation with streptavidin-horseradish peroxidase conjugate solution (diluted 1 : 1000) for 1 h at room temperature was carried out. After addition of substrate and stop solution, the amount of BDNF was determined (absorbance set in 450 nm). The standard curve demonstrates a direct relationship between optical density (OD) and BDNF concentration. Total protein was measured by Bradford's method (samples diluted 1 : 200) using bovine serum albumin (BSA) as a standard.

### 2.5. Data Analysis

Data analysis was performed using the Spearman correlation due to the fact that the dependent variables (hippocampal volume and peripheral levels of BDNF) had asymmetric distribution. Correlations between hippocampal volume, BDNF levels, working memory, and duration of disorder were also performed. All the analyses were performed considering raw volumes for the hippocampus and also for a corrected value according to total intracranial volume, due to the high variation in our age range.

SPSS 20 for Windows was used for statistical analyses. All statistical tests were two-tailed with a set at 0.05.

### 2.6. Ethical Issues

Children, adolescents, and their parents were properly informed about the goal of the project and accepted participating in the protocol and use of data anonymously for publications. This project was approved by the Ethics Committee in Research of the Hospital de Clínicas de Porto Alegre. A statement of informed consent was provided by the parent or guardian and verbal assent by the patient.

## 3. Results

During the conduction of this study, 127 patients were assessed, and 75% were excluded due to not presenting BD-I, BD-II, or BD-NOS. Two patients started the assessment but did not complete it. From the 30 patients available for the protocol, twenty-seven patients completed the entire evaluation. Three patients did not undergo MRI: one patient got pregnant, and two patients were not able due to the use of dental braces. Demographic/clinical data of the subjects are described in Table 1. The final sample was composed of 14 male and 13 female subjects, and their mean age was 13.8 years. The age of onset of bipolar disorder among patients ranged from 3 to 15 years. Intelligence quotient (IQ) in the subjects ranged around 105.77 ± 14.05. The majority of subjects presented bipolar disorder type I (77.7%), 3.7% bipolar disorder type II, and 18.6% BD-NOS. Disease duration varied from zero to 14 years (average: 4.74; SD: 3.38). From the 27 patients, 33.3% had comorbid ADHD, 18.5% had comorbid anxiety disorders, and 18.5% had formal diagnosis of ODD, as described in Table 2.

Most subjects (77.7%) were taking a number of psychiatric medications: lithium or valproate monotherapy (*n* = 4; 14.8%); atypical antipsychotics monotherapy (*n* = 4, 14.8%); combined lithium plus anticonvulsants/antipsychotics (*n* = 6, 22.2%); combined anticonvulsants/antidepressants (*n* = 1, 3.7%); multiple combination therapy (*n* = 5, 18.5%); concomitant use of stimulants (*n* = 7, 25.9%). The mean number of psychoactive medications for children in this group was 1.74.

Table 3 shows right hippocampus volume (RHV) and left hippocampus volume (LHV), respectively, 4191.05 (mm^3^) (SD = 416.30) and 4174.79 mm^3^ (SD = 506.74), as determined by automatic segmentation method. Total intracranial volume means were 1512863.14 mm^3^ (SD = 151579.76). Peripheral BDNF levels varied around 19.58 ± 6.33 (pg/*μ*g protein). The average Digit Span Total Score was 6.66. The average Digit Span Inverse Order was 4.48.

### 3.1. Primary Analysis

Table 4 shows correlation coefficients of right hippocampus volume, left hippocampus volume, and total hippocampus volume with BDNF. Those were, consecutively, 0.15, 0.03, and 0.10, with significance levels of 0.46, 0.89, and 0.62, respectively. After correction for intracranial total volume, those values were, consecutively, .090, −.125, and .007, with significance levels of .663, .544, and .972 (Table 5).

Correlation coefficients between RHV, LHV, and THV and the Digit Span Inverse Order were 0.02, 0.10, and 0.05, respectively, and are also presented in Table 4. No correlation was significant. We did not observe any correlations between BDNF levels and disorder duration, as well as in working memory as measured by the Digit Span Inverse Order test (Table 6). Adjustment for intracranial total volume has not shown a statistically significant correlation.

## 4. Discussion

In our evaluation of hippocampus volume and peripheral levels in children and adolescents with bipolar disorder, no statistically significant correlations were detected. The same occurred with respect to working memory and disease duration. We emphasize that post hoc analyses were conducted, evaluating peripheral BDNF levels and hippocampus volume in patients with higher and lower disease duration. But still no correlations were found.

Although studies in adults have been able to show the relationship between peripheral BDNF levels and hippocampus volume, we hypothesized that the lack of correlation found in this study may represent the short time of evolution of BD in children and adolescents. Usually in adult BD studies, reduced BDNF levels are found in chronic or late-stage individuals with BD, in comparison with patients in early stages of the illness [11]. The same occurs in neuroimaging studies in bipolar disorder, meaning that the effects of systemic toxicity, cognitive and functional impairment, and biological changes seen in BD tend to be cumulative and much more prominent after multiple episodes [23]. Thus, these changes may not yet be found in patients with few years of the disease, as occurs in children and adolescents with BD.

Even diseases that are proven to exert a strong influence on hippocampus morphology and structure, such as epilepsy, may still not reveal changes in neuroimaging exams when in children. For instance, studies of newly diagnosed epilepsy typically fail to find many patients with clear structural subcortical changes at the onset. Zhang et al. compared hippocampus volumes in children with temporal lobe epilepsy (TLE) and healthy controls using magnetic resonance imaging. They have not found hippocampus volume reduction in diagnosed definite/probable TLE children [24]. While there is evidence from adult studies and studies in chronic epilepsy patients that hippocampus atrophy may be a progressive lesion, there is little information regarding hippocampus abnormalities early in the course of epilepsy in patients, particularly in children [25–27].

Our study was limited, in part, by the fact that we used a convenience sample, due to the short time we had available. Therefore, our sample only offered statistical power higher than 80% to detect correlation coefficients higher than 0.5 using two-sided hypothesis tests with a significance level of 0.05. In this way, the possibility of type 2 error cannot be ruled out. Another limitation was the lack of a control group for comparison of BDNF levels, which is a suggestion to future researches. The inclusion of a control group would allow the observation of developmental differences not associated with BD. However, this is the first study addressing brain volumetrics and peripheral biomarkers in PBD, and our exploratory analyses suggest neurobiological underpinnings of BD in children and adolescents may differ from this same disorder in another developmental stage (adult life). Besides that, most of our subjects were taking psychotropic medications at the time of the assessment and were euthymic. This is not uncommon given the ethical issues inherent in discontinuing medication in children with severe psychiatric illnesses. Previous investigations demonstrated that antimanic and antidepressant agents may influence the effects of BDNF on hippocampus, and that could induce morphological changes in subcortical area in BD, which could also lead to recovery of cognitive function. Due to the cross-sectional design of our study, we cannot state that medication use may have influenced the results. Also, we ran additional analyses comparing results for patients who were on and off medication, and no significant differences have arisen [28–31].

Another limitation of our study was the use of automatic segmentation method to measure and compare hippocampal volumes. Manual and automatic segmentation methods have been compared in some studies [32–34]. The findings mostly validate the use of automatic methods for segmentation of brain structures, confirming FreeSurfer's potential to determine hippocampal volumes in large-scale studies, even though there was a systematic volume difference between FreeSurfer and manual results. Dewey et al. compared FreeSurfer and individual brain atlases using statistical parametric mapping (IBASPM) to autoassisted manual tracings [35]. They evaluated FreeSurfer to be effective for subcortical volumetry but recommend visual inspection of segmentation output along with manual correction to ensure validity of the data. Wenger et al. compared automatic versus manual segmentation in including 44 younger participants (20–30 years) and 47 older participants (60–70 years) and results reveal high stability coefficients over time for both manual and FreeSurfer segmentations [36]. With FreeSurfer, correlations over time were significantly lower in the older than in the younger age group, which was not the case with manual segmentation. Absolute agreements between the two measures, however, were considerably lower, as FreeSurfer estimated volumes to be higher. This volume difference was larger in the young than in the old. FreeSurfer detected a significant age difference in hippocampal volume, whereas manual tracing did not. However, manual tracing resulted in a significant difference between left and right Hc, whereas FreeSurfer segmented both sides in a more similar manner. They concluded that, in the younger age group, FreeSurfer can be regarded as a reliable and valid method for assessing differences in hippocampal volume, with at least as high reliability as manual tracing.

The study of possible correlations between serum levels of BDNF, working memory, and hippocampal volumetric changes in patients with BD through neuroimaging can make important contributions to the understanding of the neurobiology of these disorders, such as how and when the course of the disorder would play its effects on brain development. Our findings suggest that, in early ages, brain volumetric alterations may not be associated with the BDNF peripheral levels and cognitive dysfunction. This information raises the possibility that, in children and adolescents, BDNF level changes must not be a priority when attempting to prevent some of the future atrophic modifications.

Despite the lack of significant statistical correlation observed in this study, this is the first study in pediatric bipolar disorder correlating hippocampus volume and BDNF, and no association between these factors was observed. Replication for result confirmation is crucial, as well as interpretation of these findings in the light of a developmental context. Studies with larger samples and longitudinal studies evaluating normal and disrupted brain development, which include a control group and patients in different mood episodes, may be able to clarify the role of BDNF in brain changes caused by bipolar disorder.

## Figures and Tables

**Table 1:** Demographic data (*N* = 27).

	Mean	SD
Age (years)	13.89	2.98
Age of onset (years)	9.18	3.79
Disease duration (years)	4.74	3.38
YMRS	7.51	8.86
CDRS	28.11	11.31
QI	105.77	14.05

SD: standard deviation; YMRS: Young Mania Rating Scale; CDRS: Children's Depression Rating Scale.

IQ: intelligence quotient.

**Table 2:** Diagnostic data (*n* = 27).

	Subjects (%)
BD-I	21 (77.7%)
BD-II	1 (3.7%)
BD-NOS	5 (18.6%)
ADHD	11 (41%)
Anxiety disorders	5 (18.6%)
ODD	5 (18.5%)

**Table 3:** MRI/working memory results (*N* = 27).

	Average	Standard deviation
Right hippoc. vol^a^ (mm^3^)	4191.05	416.30
Left hippoc. vol^b^ (mm^3^)	4174.79	506.74
Total hippoc. vol^c^ (mm^3^)	8365.85	901.58
BDNF (pg/*µ*g protein)	19.58	6.33
Digit Span TS^d^	6.66	1.83
Digit Span IO^e^	4.48	1.90

^a^Right hippocampus volume; ^b^left hippocampus volume; ^c^total hippocampus volume; all were determined with automatic segmentation method.

^d^Digit Span Total Score; ^e^Digit Span Inverse Order.

**Table 4:** Correlations between levels of BDNF, hippocampal volume, and neuropsychological measures.

	RHV^a^		LHV^b^		THV^c^	
	*ρ* (rho)	P	*ρ* (rho)	P	*ρ* (rho)	P
Disease duration	.107	.595	.251	.207	.217	.276
BDNF	.148	.461	.027	.892	.100	.621
IQ	−.009	.963	.065	.749	.045	.825
Digit Span TS^f^	−.098	.587	.102	.743	.014	.985
Digit Span IO^g^	−.021	.918	.097	.632	.045	.825

^a^Right hippocampus volume; ^b^left hippocampus volume; ^c^total hippocampus volume; ^d^correlation coefficient; ^e^significance; ^f^Digit Span Total Score; ^g^Digit Span Inverse Order.

**Table 5:** Correlations between levels of BDNF and hippocampal volume controlling for intracranial volume.

	RHV^a^		LHV^b^		THV^c^	
	*ρ* (rho)	*P*	*ρ* (rho)	*P*	*ρ* (rho)	*P*
BDNF	.090	.663	−.125	.544	.007	.972

^a^Right hippocampus volume; ^b^left hippocampus volume; ^c^total hippocampus volume.

**Table 6:** Correlation among BDNF versus disease time, IQ, and digit span.

	BDNF	
	*ρ* (rho)	*P* value
Disease time	.069	.731
IQ	.069	.732
Digit span	−.187	.351
